# Generation of a large scale repertoire of Expressed Sequence Tags (ESTs) from normalised rainbow trout cDNA libraries

**DOI:** 10.1186/1471-2164-7-196

**Published:** 2006-08-03

**Authors:** Marina Govoroun, Florence Le Gac, Yann Guiguen

**Affiliations:** 1 Institut National de la Recherche Agronomique, Station Commune de Recherches en Ichtyophysiologie, Biodiversité et Environnement (SCRIBE), INRA-SCRIBE, IFR 140, Campus de Beaulieu, 35 042 Rennes Cedex, France; 2 Station INRA de Recherches avicoles, 37380 Nouzilly, France

## Abstract

**Background:**

Within the framework of a genomics project on livestock species (AGENAE), we initiated a high-throughput DNA sequencing program of Expressed Sequence Tags (ESTs) in rainbow trout, *Oncorhynchus mykiss*.

**Results:**

We constructed three cDNA libraries including one highly complex pooled-tissue library. These libraries were normalized and subtracted to reduce clone redundancy. ESTs sequences were produced, and 96 472 ESTs corresponding to high quality sequence reads were released on the international database, currently representing 42.5% of the overall sequence knowledge in this species. All these EST sequences and other publicly available ESTs in rainbow trout have been included on a publicly available Website (SIGENAE) and have been clustered into a total of 52 930 clusters of putative transcripts groups, including 24 616 singletons. 57.1% of these 52 930 clusters are represented by at least one Agenae EST and 14 343 clusters (27.1%) are only composed by Agenae ESTs. Sequence analysis also reveals that normalization and especially subtraction were effective in decreasing redundancy, and that the pooled-tissue library was representative of the initial tissue complexity.

**Conclusion:**

Due to present work on the construction of rainbow trout normalized cDNA libraries and their extensive sequencing, along with other large scale sequencing programs, rainbow trout is now one of the major fish models in term of EST sequences available in a public database, just after Zebrafish, *Danio rerio*. This information is now used for the selection of a non redundant set of clones for producing DNA micro-arrays in order to examine global gene expression.

## Background

Rainbow trout, *Oncorhynchus mykiss*, is an important fish species for aquaculture and has been introduced throughout the world. It is also probably one of the most widely studied fish species with a long history of research carried out in physiology, nutrition, ecology, genetics, pathology, carcinogenesis and toxicology (reviewed in [1]). Its relatively large size compared to model fish like zebrafish or medaka, makes rainbow trout a particularly suited alternative model to carry out biochemical and molecular studies on specific tissues or cells that are impossible to decipher in small fish models. The genomic resources in rainbow trout are now being extensively developed and a few high-throughput DNA sequencing programs of ESTs have been recently initiated [2,3]. AGENAE (Analyse du GENome des Animaux d'Elevage) [4] is a project led by the French National Institute for Agricultural Research (INRA), that focuses on genomics of several livestock species (cattle, pigs, chickens and rainbow trout). The objectives of this program are the identification and characterization of the expressed part of genomes, the mapping of entire genomes, and the study of genetic diversity in animal populations. As a first step for the characterization of the expressed part of the genome of rainbow trout, we initiated a high-throughput EST sequencing program. Among other interests, this resource will allow large scale expression profiling experiments using microarrays based on a well characterized cDNA clone collection.

## Results and discussion

### cDNA libraries construction and characterization

We constructed three directionally cloned rainbow trout cDNA libraries: two from reproductive tissues i.e., ovarian (previtellogenesis) and testicular (gonial proliferations) tissues, and one highly complex pooled tissue cDNA library. The pooled tissue library was made in order to be as representative as possible of the entire expressed genome of rainbow trout. For this purpose, mRNA from 14 different tissues (liver, kidney, adipose tissue, gills, intestine, pituitary, brain, ovary, testes, differentiating male and female gonads, muscle, interrenal and blood cells), sampled at different developmental stages or in different physiological conditions, and mRNA from entire eyed-stage embryos and hatching larvae, were used for this pooled-tissue library construction. The three resulting libraries displayed a high initial clone complexity (>1 × 10^6 ^colony-forming units). Approximately 98% of the cDNA inserts were larger than 450 bp and the average insert size ranged between 1.3 and 1.5 kb depending on the library. Each of the 3 libraries was normalized according to previously described protocols [5,6], in order to decrease the representation of abundant mRNA. All normalized libraries were subsequently submitted to one (testis library) or two (pooled-tissue library) runs of subtraction with the already sequenced clones in order to decrease redundancy.

### ESTs sequencing

High-throughput EST sequencing was carried out on these initial, normalized and subtracted libraries (Table. 1). The pooled-tissue library was the most extensively sequenced with 82% of the total sequencing effort (88 704 reads) as this library was not focused on a particular biological function, and thus of broad interest for a vast community of physiologists. The testis library was also quite extensively sequenced (13 825 reads) as this library was found to be very informative in terms of production of new sequences, while the sequencing of the ovary library was interrupted after a first round of sequencing (5 376 reads) as this library was found to be very redundant (see below in "Redundancy and quality of the libraries"). Until now, a total of 107 904 reads sequences have been performed on 84 864 clones; among those, 88.9% were found valid (96 472 sequences corresponding to high quality sequence reads of at least 100 bp with a Phred score over 20). All the valid sequences have been released in international databanks (EMBL, GenBank). The proportion of empty vectors was found to be very low.

In order to provide an important set of well annotated clones, the 5' end sequencing strategy was favoured. However, due to the use of an excess of oligo(dT) during the first reverse transcription of the library construction, the polyA sequence remained short enough to allow sequencing of the cDNA 3' ends. A 3' end sequencing strategy was therefore carried out on 23 040 (27.1%) of the sequenced clones with a good success rate (83.1% of good quality released sequences). This 3' strategy is a useful way to distinguish genes in a closely related family using the more divergent 3' end non coding region.

### Sequence analysis

#### Influence of normalization/subtraction on the pooled-tissue library

**Figure 1:**
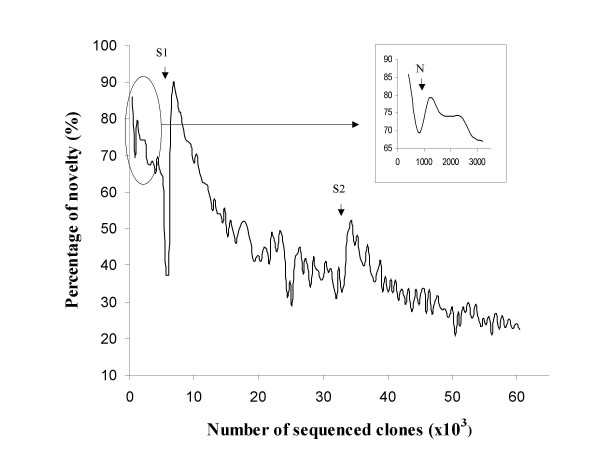
**Percentage of novel EST clusters found as a function of the number of clones sequenced in the rainbow trout pooled-tissue cDNA library**. N = Normalization. S1 = First subtraction. S2 = Second subtraction.

Following EST sequence assembly into putative transcript groups (EST clusters), we calculated the percentage of novel EST clusters as a function of the number of clones sequenced (Fig. 1). Before normalization (1 000 sequenced clones, see insert in Fig. 1), the percentage of new clusters decreased very rapidly. Normalization considerably slowed down this decrease and induced a 10% increase of novel clusters. Before the first subtraction, a few large clusters were however still observed, including a high percentage of the sequenced clones encoding putative orthologs of trypsin (for instance more than 300 ESTs for a trypsin I precursor homolog). The fact that these sequences were overrepresented in the library, even after normalization, was surprising, and we do not have any rational explanation. However, during the first subtraction we specifically removed these abundant clones by increasing their representation as driver DNA in the subtraction protocol [5]. After this subtraction, the EST analysis in the pooled-tissue library revealed that only 2 clones out of 48 800 (0.004%) belonged to these trypsin clusters. This demonstrated the efficiency of the specific subtraction strategy in decreasing redundancy. As a mater of fact, the gain of novelty was then rather high following the first subtraction (55%), probably due to the removal of these largest clusters. Following the second subtraction the gain in novelty was around 15–20%.

#### Redundancy and quality of the libraries

**Figure 2:**
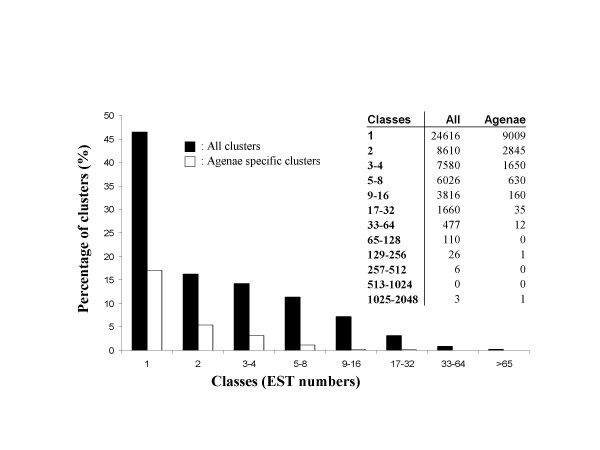
**Histogram of cluster sizes**. Repartition in the different cluster size classes of the complete collection of trout clusters (black squares) and of Agenae specific clusters (open squares), both expressed in proportion of all clusters, based on Sigenae rainbow trout EST clustering version 3. The table represented the number of clusters in each cluster size class.

With regards to clone redundancy, we listed the 20 biggest clusters considering all publicly available ESTs (Table. 2), and the 20 biggest clusters containing only Agenae ESTs (Table. 3). Among these clusters, the 2 biggest ones correspond to ESTs which are highly represented in (BX072800.1.p.om.3) or specific to (CR361581.1.p.om.3) the Agenae ovary library. The best Swissprot hit for cluster BX072800.1.p.om.3 corresponds to a zona pellucida sperm-binding protein precursor, a protein that is known to be highly expressed in the ovary [7] and whose cDNA has already been described as over-represented in a fish ovary library [8]. Cluster CR361581.1.p.om.3 does not exhibit any significant homology and a careful examination of the ESTs belonging to this cluster show that 98.5% of these ESTs actually start at exactly the same nucleotide position, which probably reflects an amplification artefact that occurred during the process of library construction. Similarly, 10 out of the 20 biggest clusters of ESTs specific to the Agenae libraries are also either specific to, or overrepresented in this ovary library (Table. 3). Due to this rather poor quality in terms of novel sequence discovery, we stopped sequencing this library. Apart from these highly redundant ESTs from the ovary library, redundancy was found to be relatively low in other Agenae libraries with the vast majority of the EST sequences within cluster classes containing less than 33 ESTs (Fig. 2). As shown in table 2, the pooled-tissue library produced 2 of the biggest trout clusters. One cluster (CA369471.1.p.om.3; 389 ESTs) corresponds to a homolog of the zebrafish protein ES1 that is specifically expressed in the adult retina [9]. The other (CA368365.1.p.om.3; 342 ESTs) is one of the large clusters of putative orthologs of trypsin overrepresented in the non subtracted pooled-tissue library mentioned above. No highly redundant clusters of ESTs were identified in the testis library with the largest clusters represented by only 4 ESTs (CR362356.1.p.om.3, CR370007.1.p.om.3 and CR365721.1.p.om.3). This demonstrated the high quality of this testis library in terms of diversity and probably reflects the high complexity of the repertoire of genes expressed in testicular tissue as previously shown in large scale ESTs sequencing programs in other fish species [8,10]

#### Contribution of the Agenae EST collections

**Figure 3:**
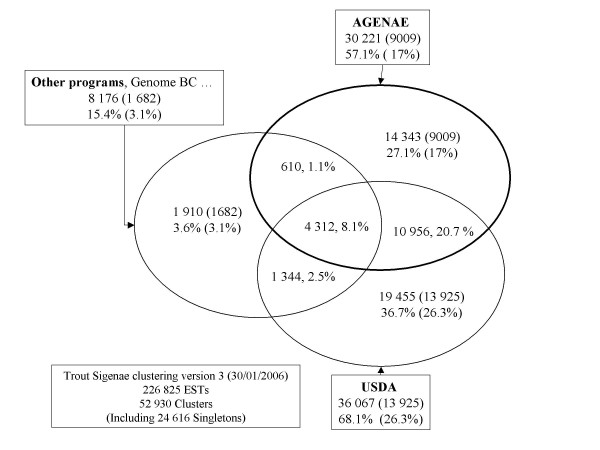
**Diagram showing the number and the relative proportion (%) of shared and unique clusters**. Shared clusters contain ESTs from different projects (AGENAE, USDA, Others) and unique clusters only contain ESTs originating from one project. Clusters are made of one (called singletons and represented under brackets) or more ESTs assembled together following clustering analysis. USDA libraries were 01 to 10RT – NCCCWA libraries and 115RT – NCCCWA library.

The overall released data, including sequences from the testis and ovary libraries, represented 42.5 % of the total rainbow trout sequences (96 472 over 226 825) in international databases in February 2006. Based on the SIGENAE trout assembly version 3 [11], our EST sequences added 14 343 unique clusters (27.1%) to the total of 52 930 clusters of putative transcript groups characterized after clustering of all ESTs available in rainbow trout (Fig. 3). Among these 52 930 clusters (including 24 616 Singletons), 30 221 (57.1 %) were represented by at least one Agenae EST. These figures were obtained by running CAP assemblies under default parameters (our values were: at least 75 bp with 96 % similarity), and are close to those found in the TIGR clustering. However, we do realise that the number of contigs (52 930) can vary when different stringencies are used, and that when using the default parameters there may be cases where paralogous genes and certainly alleles are clustered, although we have not yet gone deeply into that subject. The UNIGENE cluster number is smaller (32 400), may be in part because it contains more paralogous gene clustering, but more likely because it does not use a large part of the singletons available.

### Sequence annotation

For 12 out of the 14 single organs initially gathered to construct the pooled-tissue library, a rapid search identified at least one EST matching for a gene considered as "specifically" expressed in this tissue (Table. 4). This shows that this library was potentially representative of the various tissues used, although we do realize that some of these cited genes are probably not strictly tissue specific but could be better described as genes which are highly expressed in a particular tissue. Low abundance cDNA may be difficult to detect through such EST sequencing projects. However, the fact that most genes are expressed in many tissues, combined with the normalization procedure probably increased the chances of picking them up in such a pooled-tissue library.

Although cDNA library construction and EST sequencing is a time and money consuming task, the most common strategy still consists in sequencing numerous tissue specific libraries in order to provide a large number of clusters. For instance, in the medaka, *Oryzias latipes*, 26 689 clusters were generated from 147 802 EST obtained from 29 different tissue specific cDNA libraries (TIGR gene Index, Release 5.0, May 17, 2004). In trout, with slightly more ESTs (157 116) coming mainly from 2 pooled-tissue libraries (AGENAE and 1RT-NCCCWA USDA), the last TIGR clustering (Release 5.0, January 31, 2005) detected twice as many clusters (50 773). The pooled-tissue libraries strategy combined with normalization/subtraction methods may thus be a better approach for enrichment of different transcripts. Actually, some recent EST projects rely on pooled-tissue libraries [2,12,13]. However the pooled-tissue strategy suffers from a lack of information concerning mRNA tissue origin and it is then not possible to carry out *in silico *analysis of tissue differential expression [14]. A strategy based on pooled-tissue library with a tissue specific DNA identification tag, has recently been proposed [13]. This would combine the advantages of the pooled-tissue library with keeping the information on the tissue origin of each EST.

## Conclusion

In conclusion our rainbow trout cDNA libraries provided a large set of well characterized clones for future studies. The Agenae sequencing project together with ongoing collaborative efforts of the ARS-USDA program [2] and the Genome BC project [3] now places rainbow trout in the position of being one of the major fish models, in terms of ESTs present, in public databases just after the zebrafish, *Danio rerio *(for instance 24 466 clusters in UniGene Build #17 09 Feb 2006 for rainbow trout and 32 400 clusters for zebrafish in UniGene Build #89, 05 Dec 2005). We are now using this important sequence information and our corresponding clone collections for producing DNA arrays in order to examine global gene expression in rainbow trout [15,16]. A micro-array, containing 9 000 well annotated and unique cDNAs chosen for their informative annotation and their low redundancy, is now produced in large numbers in our resource centre (CRB-GADIE) [17], and used for gene expression profiling by several research teams.

## Methods

### Tissues samples and RNA preparation

Research involving animal experimentation has been approved by the authors' institution (authorization number 35-14) and conforms to the principles for the use and care of laboratory animals in compliance with French and European regulations on animal welfare. Rainbow trout were obtained from the Drennec experimental farm (Drennec, France). For the pooled-tissue cDNA library, more than 30 different individual fish of both sexes, issued from 3 different strains (autumnal, spring and winter spawning strains) were used; these strains themselves originated from at least 3 different French or Belgium regions. The following tissues, obtained at different stages of their development for several of them, were sampled and stored at -80°C before RNA purification: liver, kidney, adipose tissue, gills, intestine, pituitary, brain, ovary, testes, early differentiating male and female gonads, muscle, interrenal, leucocytes, blastula embryos, eyed-stage and hatching larvae, skin and blood cells. For the testis and ovary libraries, testes contained only spermatogonia (Stage I and II according to Billard's classification [18] ), and the ovary was at the previtellogenesis stage. Total RNA was extracted from each frozen tissue using TRIzol^® ^reagent (Gibco BRL, Gaithersburg, MD). The quality of total RNA was first checked by electrophoresis on a 1% agarose gel, then by a reverse transcription test using trace amounts of [α-32P] dCTP [19]. The radioactive cDNA obtained was analyzed by autoradiography after electrophoresis on a denaturing alkaline agarose gel. Some total RNA samples (originating from blastula embryos, leucocytes, and skin) were found to be unsuitable for oligo(dT) primed reverse transcription and were not incorporated into the pool of total RNA used for the final construction of the pooled-tissue cDNA library. Total RNAs from the 14 tissues (liver, kidney, adipose tissue, gills, pituitary, brain, ovary, testes, differentiating gonads, muscles, intestine, interrenal and blood cells) plus entire eyed stage embryo, and hatching larvae RNAs were pooled in equal proportions. Poly-A-selected mRNA was prepared by purification of pooled total RNA on a oligo(dT) – cellulose column as previously described [19]. Quality of mRNA purification was checked by electrophoresis of a small aliquot on 1% agarose and by a reverse transcription test using trace amounts of [α-32P] dCTP.

### Library construction

cDNA libraries were constructed in the pT7T3-Pac vector as initially described by B. Soares, M. Bonaldo and collaborators [5,6,19]. Briefly, starting from the mRNA, cDNA synthesis was carried out with a NotI-dT18 primer to allow directional cloning. After size selection chromatography (≥ 500 bp), the double-stranded cDNA were ligated to EcoRI adapters, digested with NotI, and directionally cloned into the NotI and EcoRI digested pT7T3-Pac vector. The library was electroporated and then amplified in DH10B competent cells (Invitrogen). Normalization and subtraction was carried out according to [19]. Briefly, single strand DNA circles were produced from the directional cDNA libraries (tester DNA). These single strand DNA circles were also used to produce doubled strands DNA (driver DNA) corresponding to the inserts, by PCR using vector primers T7 and T3, flanking the insertion sites. Tester DNA was then melted and reannealed with an excess of driver DNA and the remaining single strand driver DNA (normalized or subtracted library) was then purified by hydroxyapatite chromatography. These single strands DNA molecules were then converted to partial duplex by random priming and electroporated into bacteria to produce the final normalized or subtracted library (see [19] for additional details).

The cDNA mean insert sizes of the libraries have been estimated on 50 individual clones by PCR using T3 and T7 as primers flanking the inserts.

### Sequencing

The libraries were plated onto 2xYT medium and arrayed robotically into 96 well plates at the INRA National Biological Resources Centre for Animal Genomics (CRB GADIE) [17]. Plates were then sent to a sequencing company [20], and bacterial clones were sequenced following plasmid DNA purification with T7 primer for 5' end sequencing and T3 primer for 3' end sequencing.

### Sequence analysis and EST clustering

EST sequences were cleaned from vector and adaptor sequences and sequences containing contaminants such as E. Coli, Yeast, Mitochondria, Ribosome or Univec were removed from the analysis. Only sequences with a PHRED score over 20 on at least 100 bp were released in the EST division of the EMBL-EBI (European Molecular Biology Laboratory – European Bioinformatic Institute) Nucleotide Sequence Database. The calculation of the redundancy and proportion of clusters generated by the different EST sequencing projects was carried out using the SIGENAE trout clustering version V3 [11]. The percentage of novelty in the pooled-tissue cDNA library was calculated as follows: knowing that some clones have been sequenced at both ends (5' and 3') one representative sequence was selected for each clone (the selected sequence was the 3' end if it existed). Then the clones were ordered by name for each block of clones [using an incremental step of 400 clones] and the number of clusters was counted. The figures shown in the graph are therefore the number of new clusters generated by the 400 next sequenced clones. This work was done using an R [21] home made routine extracting data from a PostgreSQL database. Sequences corresponding to putative "tissue specific" proteins in the pooled-tissue cDNA library were found using a best blast hit strategy for the approximation of orthologs rainbow trout ESTs. Tissue specific proteins were chosen according to their description as "tissue specific" in the literature and their amino-acid sequence was used to search at NCBI [22] for a putative orthologs in rainbow trout using a TblastN algorithm on Database "EST-others" with a query limited by the term "Oncorhynchus" and other parameters set to default. The best hit sequence was then double checked by a blastx query on a non-redundant Database. For already known tissue specific genes in rainbow trout a blastn query was carried out and EST sequences showing 100% identity were selected.

## Authors' contributions

MG carried out most of the work needed for construction and normalization of the cDNA libraries with substantial help from FLG and YG. FLG and YG conceived the study, participated in its design and coordination. YG also drafted the manuscript. All authors read and approved the final manuscript.

## Figures and Tables

**Table 1:** Summary of the numbers of sequenced and released ESTs in the different AGENAE trout cDNA libraries.

Libraries	pooled-tissue	Testis	Ovary	Total
Number of sequenced Clones	65 664	13 824	5 376	84 864
Number of Sequences	88 704	13 824	5 376	107 904
Including 5'	65 664	13 824	5 376	84 864
Including 3'	23 040	0	0	23 040
Published sequences	79 037	12 499	4 936	96 472

**Table 2:** Top 20 most redundant EST clusters.

Cluster Name	Cluster depth	Best swissprot hit	Hit description	Over-expressed in Agenae libraries
BX072800.1.p.om.3	1463	P48831	Zona pellucida sperm-binding protein 3 precursor	Yes (Ovary)
CR361581.1.p.om.3	1460	NULL		Yes (Ovary)
CA352033.1.p.om.3	1434	P10995	Actin, alpha sarcomeric/cardiac (Actin alpha 2)	No
BX073087.1.p.om.3	431	P21993	Prolactin precursor (PRL)	No
CA342041.1.p.om.3	402	P02141	Hemoglobin beta-4 subunit	No
CA369471.1.p.om.3	389	P30042	ES1 protein homolog, mitochondrial precursor (Protein KNP-I)	Yes (Pooled-Tissue)
CA368365.1.p.om.3	342	P35031	Trypsin I precursor	Yes (Pooled-Tissue)
BX074651.1.p.om.3	314	P20332	Somatotropin 2 precursor (Growth hormone 2)	No
CA341574.1.p.om.3	264	P35979	60S ribosomal protein L12	No
CA343108.1.p.om.3	255	P02609	Myosin regulatory light chain 2, skeletal muscle isoform (G2)	No
BX073970.1.p.om.3	224	P07064	Somatotropin precursor (Growth hormone)	No
CA341678.1.p.om.3	197	P70083	Sarcoplasmic/endoplasmic reticulum calcium ATPase 1	No
CA341906.1.p.om.3	178	Q90YV7	60S ribosomal protein L11	No
CA341950.1.p.om.3	173	Q90Z10	60S ribosomal protein L13	No
CA341578.1.p.om.3	170	P0AFB7	Nitrogen regulation protein NR(II)	No
CA345253.1.p.om.3	161	P47954	Glutathione S-transferase P	No
CA341772.1.p.om.3	159	O57561	60S ribosomal protein L18a	No
CA342754.1.p.om.3	157	Q91487	60S ribosomal protein L13a (Transplantation antigen P198 homolog)	No
CA342547.1.p.om.3	155	P02457	Collagen alpha 1(I) chain precursor	No
CA378499.1.p.om.3	153	NULL		No

The 20 most redundant EST clusters in all rainbow trout cDNA libraries are listed with their Sigenae cluster name and the number of ESTs within each cluster (cluster depth). When a homology search using blastx was carried out against the Swissprot database returned a significant homology (blast score > 100), the accession number of this best putative homolog is given along with its associated description. When a cluster contains an over-representation of ESTs found only in one Agenae library, the name of this library is given in the last column.

**Table 3:** Top 20 most redundant Agenae specific EST clusters.

Cluster Name	Cluster depth	Best swissprot hit	Hit_description	Over-expressed in a specific Agenae library
CR361581.1.p.om.3	1460	NULL		Yes (Ovary)
CR361588.1.p.om.3	139	NULL		Yes (Ovary)
BX304386.1.p.om.3	63	Q99PP2	Zinc finger protein 318 (Testicular zinc finger protein)	Yes (Ovary)
BX871489.1.p.om.3	59	NULL		Yes (Ovary)
BX310674.1.p.om.3	49	NULL		No
CR361690.1.p.om.3	48	Q91YT0	NADH-ubiquinone oxidoreductase 51 kDa subunit	Yes (Ovary)
BX300811.1.p.om.3	48	Q94480	VEG136 protein (Fragment)	Yes (Pooled-Tissue)
BX304242.1.p.om.3	40	P98165	Very low-density lipoprotein receptor precursor	No
BX310459.1.p.om.3	37	Q9ESN4	Complement C1q-like protein 3 precursor (Gliacolin)	No
CR361738.1.p.om.3	36	NULL		Yes (Ovary)
BX300472.1.p.om.3	36	NULL		No
CR361612.1.p.om.3	33	Q8N6G5	Chondroitin beta-1, 4-N-acetylgalactosaminyltransferase 2	Yes (Ovary)
CR361716.1.p.om.3	33	P28570	Sodium- and chloride-dependent creatine transporter 1	Yes (Ovary)
BX860653.1.p.om.3	33	NULL		Yes (Ovary)
BX321717.1.p.om.3	32	P50416	Carnitine O-palmitoyltransferase I, mitochondrial liver isoform	No
BX304069.1.p.om.3	31	Q9NR09	Baculoviral IAP repeat-containing protein 6	No
BX321042.1.p.om.3	30	Q9R190	Metastasis-associated protein MTA2	Yes (Pooled
BX299242.1.p.om.3	28	O08849	Regulator of G-protein signaling 2 (RGS2)	Yes (Pooled
BX317551.1.p.om.3	28	Q9WYK0	Endonuclease III (DNA-(apurinic or apyrimidinic site) lyase)	Yes (Ovary)
BX298697.1.p.om.3	28	NULL		Yes (Pooled-Tissue)

The 20 most redundant EST clusters composed of only ESTs originated from Agenae rainbow trout cDNA libraries are listed with their Sigenae cluster name and the number of ESTs within each cluster (cluster depth). When homology search using blastx against the Swissprot database returned a significant homology (blast score > 100) the accession number of this best putative homolog is given along with its associated description. When a cluster contains an over-representation of ESTs found in only one Agenae library, the name of this library is given in the last column.

**Table 4:** Tissue representation in the pooled-tissue cDNA library.

Tissues	Protein	Species	References	TBLASTN	BLASTX	Sequence ID
Testis	Testis Creatine kinase	*O.mykiss*	[23]	0	/	tcaa0001c.e.17 (BX073510)
Ovary	Factor in germ line alpha (Figa)	*M. musculus*	[24]	89, 7e-19	89, 9e-17	tcad0001a.a.19 (BX074063–BX074064)
Adipose tissue	Perilipin (PLIN)	*H. sapiens*	[25]	197, 7e-51	197, 2e-49	tcbk0010c.i.14 (BX872787)
Kidney	Collectrin	*H. sapiens*	[26]	141, 1e-34	141, 1e-32	tcay0014b.i.05 (BX304379–BX304380)
Fetal gonads	DMRT1	*O.mykiss*	[27]	0	/	tcad0009a.n.11 (BX081098–BX081099)
Liver	Liver-basic fatty acid binding protein	*D. rerio*	[28]	222, 1e-59	222, 3e-57	tcay0037b.e.14 (BX319796–BX319797)
Gills	FHL5	*A. japonica*	[29]	399, 1e-112	404, 1e-117	tcad0002a.a.17 (BX074365)
Pituitary	Growth Hormone factor 1 (Pit-1)	*O. keta*	[30]	156, 4e-40	156, 2e-38	tcay0007b.j.13 (BX301445–BX301446)
Blood	ERMAP	*H. sapiens*	[31]	163, 1e-40	163, 9e-43	tcbk0052c.l.01 (BX885983)
Brain	Brain cell membrane protein 1 (BCMP1)	*C. familiaris*	[32]	269, 3e-73	247, 2e-64	tcbk0029c.j.07 (BX885584)
Intestine	Intestinal mucin-like peptide	*R. Norvegicus*	[33]	217, 2e-56	194, 1e-48	tcac0004c.g.02 (BX083186–BX083187)
Muscle	Muscle LIM protein	*R. Norvegicus*	[34]	315, 4e-97	270, 2e-71	tcba0018c.g.07 (BX861190)

Representative "tissue specific" protein homologs in the pooled-tissue cDNA library. Clone identity (clone ID) is given in the sequence ID column with the GenBank accession numbers in brackets. When more than one sequence was carried out on one clone (5' and 3' EST sequences), the second accession number is noted -X. For rainbow trout EST matching a rainbow trout gene only a blastN strategy was used.
